# Molecular cloning and functional analysis of a *Chrysanthemum vestitum GME* homolog that enhances drought tolerance in transgenic tobacco

**DOI:** 10.1038/s41598-022-17815-7

**Published:** 2022-08-08

**Authors:** Jingjing Li, Hongyuan Xu, Xiaoyu Li, Lijun Wang, Xuan Wang, Yanqing Liu, Yueping Ma

**Affiliations:** College of Life and Health Sciences, Northeastern University, Shenyang, 110819 China

**Keywords:** Biochemistry, Biotechnology, Developmental biology, Molecular biology, Plant sciences

## Abstract

GDP-mannose 3, 5-epimerase (GME, EC 5.1.3.18), a key enzyme in the ascorbic acid synthesis pathway, catalyzes the conversion of GDP-D-mannose to GDP-l-galactose in higher plants. Here, a homolog of *GME* was isolated from *Chrysanthemum vestitum*. The cDNA sequence of *CvGME* was 1131 bp and contained a complete open reading frame encoding a protein comprising 376 amino acids. Quantitative real-time PCR analysis revealed that *CvGME* was most highly expressed in the stems and roots. Phylogenetic analysis showed that CvGME was closely related to LsGME from *Lactuca sativa*. Subcellular localization studies revealed that CvGME was localized in the nucleus. Heterologous expression of *CvGME* in transgenic tobacco plants increased the ascorbic acid content in the leaves. In addition, overexpression of *CvGME* reduced the malondialdehyde content and increased superoxide dismutase and peroxidase activity in tobacco leaves compared to those in the wild-type plants under drought stress conditions, explaining the increased drought tolerance of transgenic tobacco lines. These results suggest that *CvGME* can effectively enhance the tolerance of plants to drought by increasing the ascorbic acid content, which may help improve the drought tolerance of chrysanthemums through molecular breeding.

## Introduction

Chrysanthemums, a traditional Chinese flower, is one of the four largest cut flower varieties in the world due to its great ornamental and economic value^1,2^. During the long-term cultivation process, the stress resistance of chrysanthemums gradually decreases; for example, it becomes less tolerant to drought or waterlogging. Thus, the production and commercialization of chrysanthemum have been greatly restricted^3,4^. Drought stress has become one of the main factors limiting chrysanthemum production and landscaping applications, largely due to global water shortages^5,6^. Development of new chrysanthemum cultivars with increased drought stress tolerance is therefore a key goal of chrysanthemum breeders^7^. Conventional breeding approaches such as crossbreeding and mutation breeding are often laborious and ineffective. Recently, genetic transformation techniques have become powerful tools for improving abiotic stress tolerance^8,9^.

Ascorbic acid (AsA), also known as vitamin C, is a pivotal antioxidant in plants^10–12^. Many studies have shown that AsA plays an important role in plant development and plant tolerance to various environmental stressors^13–16^. Additionally, humans and some other animals cannot synthesize vitamin C, with food being the main source, especially, ascorbate derived from plants^17^.

GDP-D-mannose-3,5-epimerase (GME) is a key enzyme in the AsA synthesis pathway, which catalyzes the conversion of GDP-D-mannose to GDP-l-galactose^13,18,19^. Previous studies have shown that *GME* expression levels are positively correlated with the AsA content^20,21^. Overexpression of *GME* homolog enhances plant tolerance to abiotic stress with AsA accumulation^22–25^. Therefore, modulating *GME* expression is a novel strategy for regulating the AsA content and alleviating abiotic stress.

To date, the *GMEs* have been cloned from a number of plants including alfalfa, tomato and kiwifruit^22,23,26^. However, there are few reports on the *GME* in chrysanthemums. *Chrysanthemum vestitum* is a wild parental species of chrysanthemums^27,28^. In this study, a *GME* homolog was isolated from *C. vestitum*, and overexpressed in tobacco, a model organism for studying plant gene expression. The effects of *CvGME* on drought tolerance in transgenic tobacco were investigated to ascertain its importance in AsA synthesis and enhanced stress resistance in plants.

## Results

### Cloning of the *CvGME*

**Figure 1:**
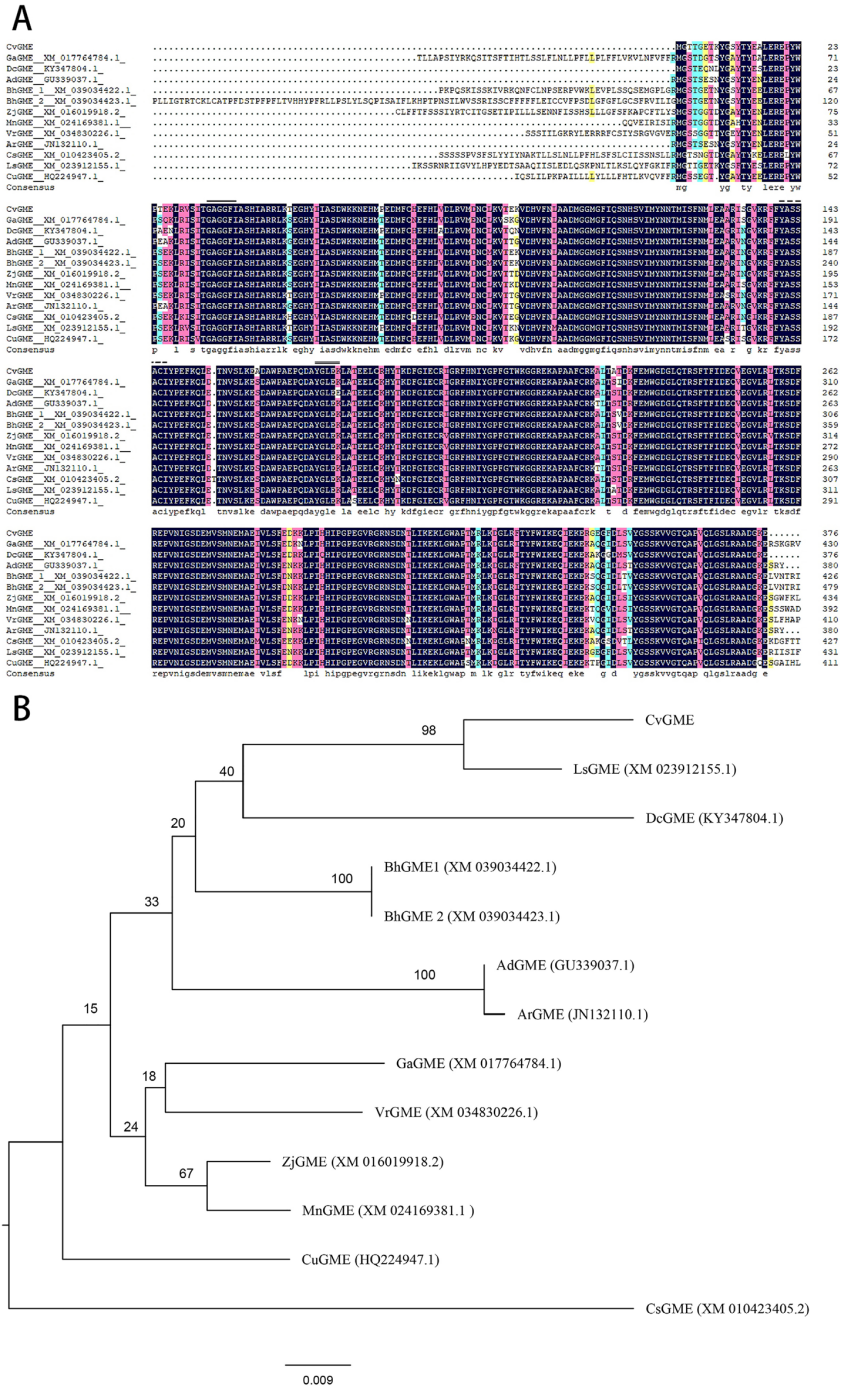
Comparison of CvGME with GME homologs. (**A**) Amino acid sequence alignment of CvGME and GME homologs in other plant species; the single black solid line (GAGGFI) represents the NAD binding site. The single black dotted line (YASSACI) represents serine (Ser). The double solid line (YGLEK) represents enzyme catalytic site (YxxxK). (**B**) Phylogenetic analysis results of the amino acid sequences. CvGME, *Chrysanthemum vestitum*; LsGME, *Lactuca sativa* (XM_023912155.1); DcGME*, Daucus carota* (KY347804.1); BhGME1, *Benincasa hispida* (XM_039034422.1); BnGME2, *Benincasa hispida* (XM_039034423.1); AdGME, *Actinidia deliciosa* (GU339037.1); ArGME, *Actinidia rufa* (JN132110.1); GaGME, *Gossypium arboretum* (XM_017764784.1); VrGME, *Vitis riparia* (XM_034830226.1); ZjGME*, Ziziphus jujuba* (XM_016019918.2); MnGME*, Morus notabilis* (XM_024169381.1); CuGME, *Citrus unshiu* (HQ224947.1); and CsGME, *Camelina sativa* (XM_010423405.2).

The complete cDNA of the *GME* homolog gene was successfully amplified from the leaves of *C. vestitum*. The length of the sequence was 1131 bp, and the encoded protein consisted of 376 amino acids, containing an NAD-binding domain, tryptophan domain, and a short-chain dehydrogenase catalytic domain (GenBank accession numbers OL962692, named *CvGME*). *CvGME* contains five introns with positions conserved in most other *GME* homolog genes (OM304347). These results suggest that the *GME* homolog was isolated from *C. vestitum*. Comparison of the putative amino acid sequences of CvGME with other GME homologs revealed that sequence identity ranged from 81.25 to 89.11% and the highest identity of 89.11% was shared with LsGME from *Lactuca sativa* (Fig. 1A). Phylogenetic analysis based on *GME* predicted amino acid sequences showed that CvGME was clustered with LsGME into a clade with a 98% bootstrap support value consistent with biological evolutionary patterns (Fig. 1B).

### Expression of *CvGME* in *Chrysanthemum vestitum*

**Figure 2:**
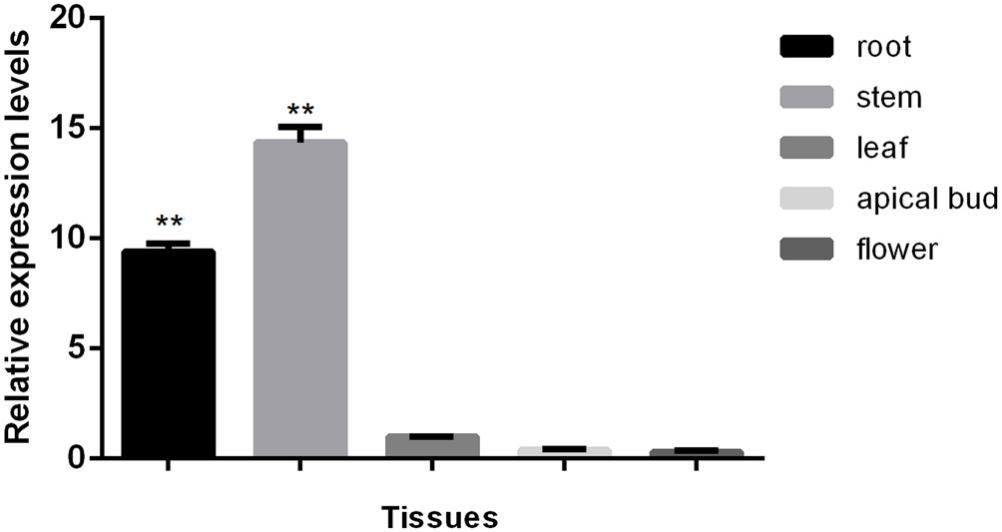
*CvGME* expression in various tissues isolated from *C. vestitum*. Data showed as mean ± SD of three independent experiments. Asterisks indicate values that are significantly different from those of leaf (*p* < 0.01).

The expression pattern of *CvGME* in different tissues of *C. vestitum* was explored using quantitative real-time polymerase chain reaction (qRT-PCR). The results showed that *CvGME* was expressed at low levels in the flower and apical bud, whereas it was highly expressed in the stem and root (Fig. 2).

### CvGME is located in the nucleus

**Figure 3:**
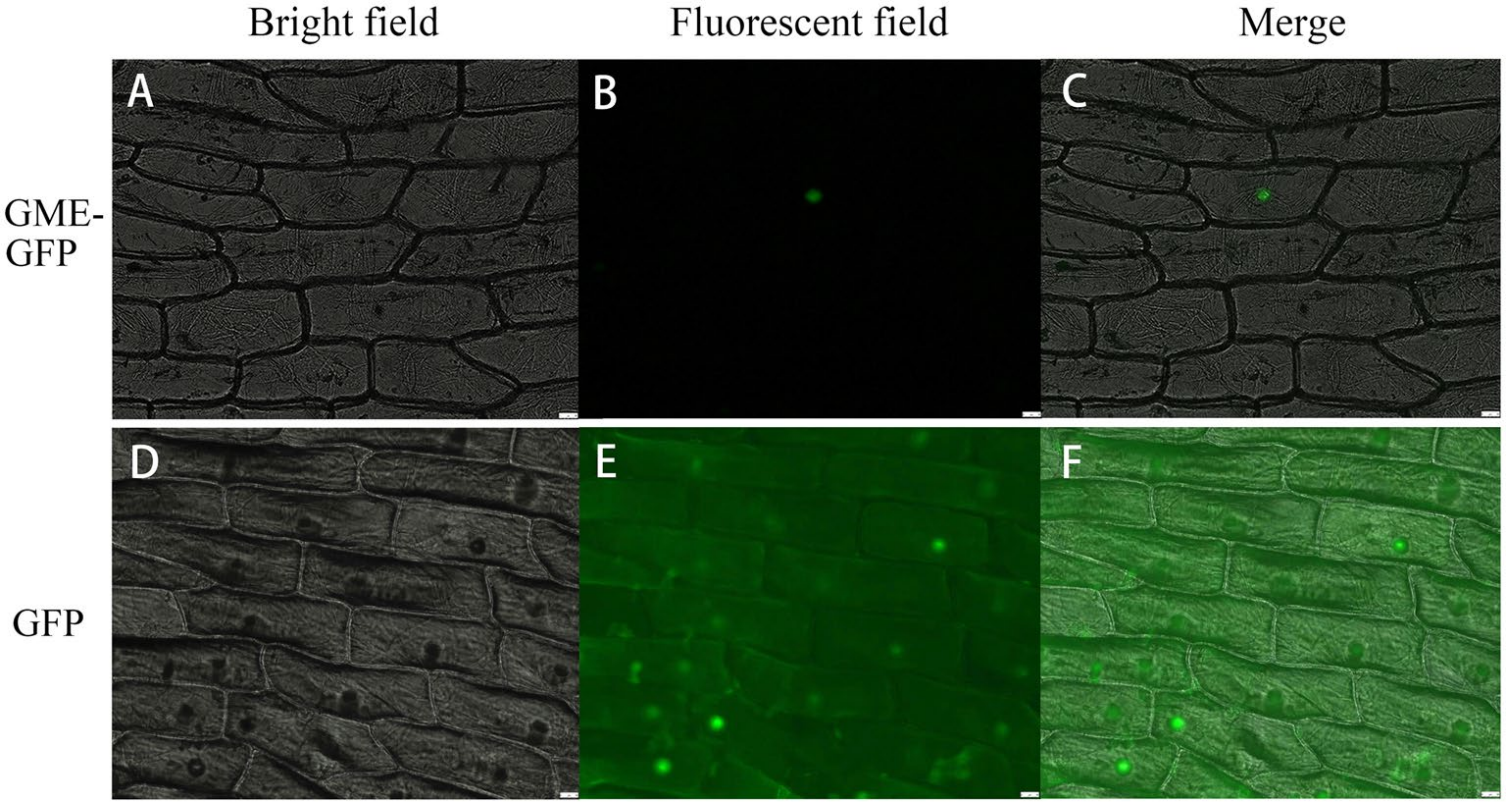
Subcellular localization of GME. GFP-tagged GME protein was transiently expressed in onion epidermal cells. (**A**– GME-GFP; (**D**–**F**) GFP. Left to right: bright field, fluorescent field, and merged image.

*CvGME*-*GFP* was transiently expressed in onion epidermal cells to confirm the subcellular localization of *CvGME*. The GME-GFP fluorescent signal was localized only in the nucleus (Fig. 3A–C), whereas the -GFP fluorescent signal was observed in the nucleus, cytoplasm, and cell membrane (Fig. 3D–F).

### Ectopic expression of *CvGME* in tobacco

**Figure 4:**
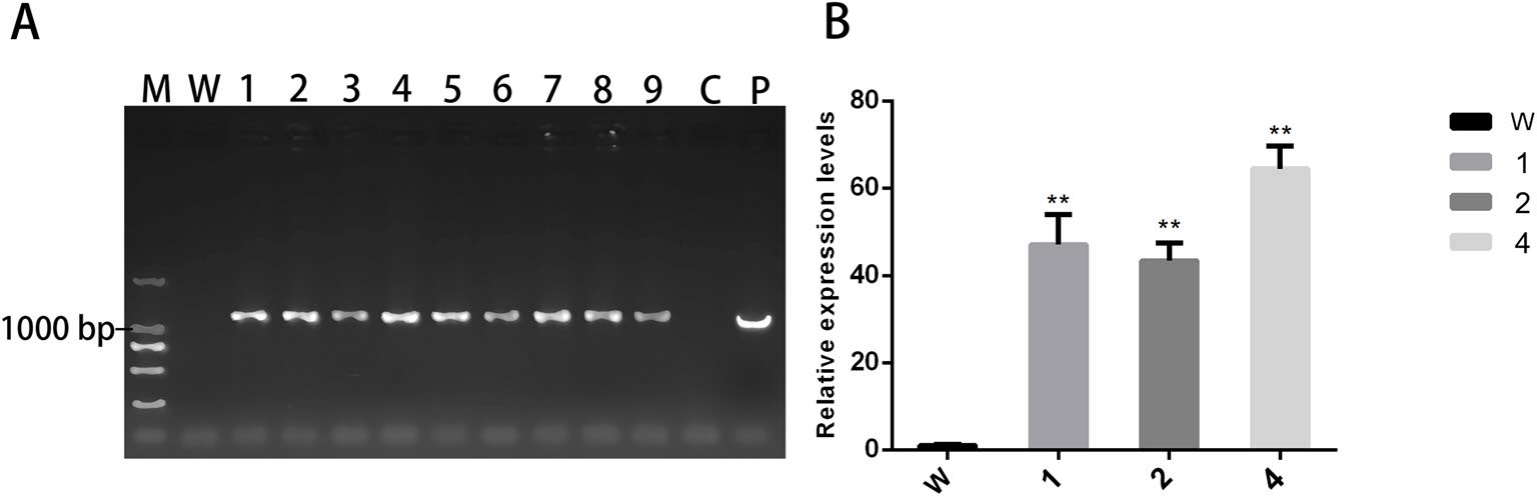
Transgenic tobacco identification using (**A**) genomic PCR, M: DL2000 marker, W: wild-type tobacco, 1–9: transgenic tobacco lines, C: blank control, P: positive control. (**B**) *CvGME* expression in wild-type and transgenic tobacco line 1, 2 and 4, W: wild-type tobacco; 1, 2 and 4: T1 generation of three different transgenic lines. Data showed as mean ± SD of three independent experiments. Asterisks indicate values that are significantly different from those of the wild-type plants (*p* < 0.01).

Approximately 60 independent transgenic tobacco plants were obtained after rooting on Murashige and Skoog (MS) medium containing kanamycin and rifampicin. All plants were confirmed to be *CvGME* transgenic using genomic PCR (Fig. 4A) and qRT-PCR (Fig. 4B). Transgenic lines with high levels of transgene expression were selected for further analysis. The *CvGME* overexpressing transgenic tobacco plant lines 1, 2, and 4 showed 48.6, 43.4, and 64.4 fold upregulated expression compared with the wild-type tobacco plant, respectively.

### *CvGME* improved tolerance to drought stress

**Figure 5:**
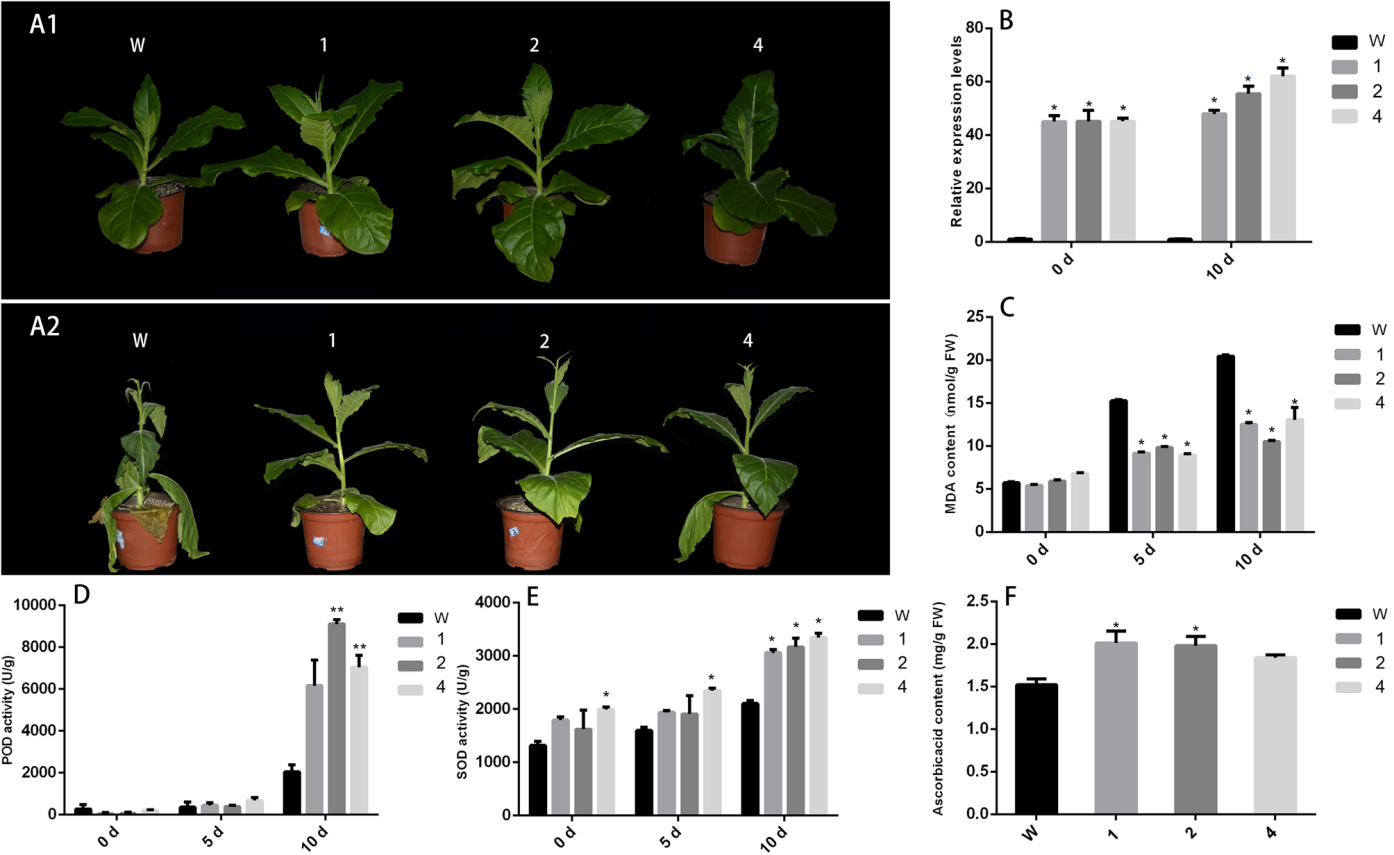
Analysis of drought resistance in wild-type and *CvGME* transgenic plants. (**A1**) Plants before drought stress treatment. (**A2**) Tobacco plants under drought stress for 10 days. W: wild-type tobacco; 1, 2 and 4: T1 generation of three different transgenic lines. (**B**) *CvGME* expression in tobacco after 10 days drought treatment. (**C**) MDA content. (**D**) Peroxidase (POD) activity. (**E**) Superoxide dismutase (SOD) activity. (**F**) AsA content of wild-type and *CvGME* transgenic plants. Data showed as mean ± SD of three independent experiments. Asterisks indicate values that are significantly different from those of the wild-type plants (**p < 0.01 or *p < 0.05).

Four-week-old T1 generation transgenic tobacco lines 1, 2, and 4 were subjected to drought condition for 10 days to evaluate the function of *CvGME* in drought stress tolerance. Under drought stress treatment, the transgenic lines grew well, whereas wild-type tobacco exhibited yellowing and wilting earlier than its transgenic counterparts (Fig. 5A1,A2). When watering was resumed, the transgenic tobacco plants recovered quickly, with healthy leaves appearing gradually from bottom to top. However, the recovery process of wild-type tobacco was slow, with only the young top leaves recovering.

The transcript levels of *CvGME* in response to drought stress were measured using qRT-PCR. Compared with that in the wild plants, *CvGME* expression in transgenic tobacco significantly increased after 10 days of drought treatment (Fig. 5B).

The malondialdehyde (MDA) content was not observably different between the wild-type and transgenic plants under normal condition. However, after drought treatment, the MDA content gradually increased in all plants with the duration of stress, but a considerably greater increase rate was observed in the wild-type plants than in the transgenic plants. After 5 days of exposure to drought stress, the MDA content in the wild-type plants was 1.66, 1.7, and 1.55 times the level in *CvGME* transgenic tobacco plant lines 1, 2, and 4, respectively. After 10 days of drought stress, MDA content of the wild-type plant was 1.63, 1.94, and 1.56 times that in the transgenic plants (Fig. 5C).

There is no significant differences in peroxidase (POD) activities between the wild-type and transgenic tobacco plants under normal conditions. The superoxide dismutase (SOD) activity in the leaves of transgenic plants was slightly higher than in the wild-type plants before drought treatment. After drought stress treatment for 5 days, POD activity in the leaves of 1, 2, and 4 transgenic plants was 1.21, 1.19, and 1.46 times higher than that in the leaves of wild-type plants, respectively. SOD activity in the leaves of 1, 2, and 4 transgenic plants was 1.21, 0.98 and 1.22 times higher than that in the wild-type plants, respectively. After 10 days of exposure to the drought treatment, the POD activity in leaves of transgenic plants was 3.01, 4.45, and 3.44 times higher than in the wild-type tobacco, and the SOD activity in the leaves of 1, 2, and 4 transgenic plants was 1.45, 1.03, and 1.05 times higher than that in the wild-type plants, respectively. Thus, the POD activity of the transgenic lines was significantly higher than that in the wild-type plant (Fig. 5D,E). These results demonstrate that overexpression of *CvGME* in tobacco increases its tolerance to drought stress.

### *CvGME* improved AsA content in transgenic tobacco

The content of AsA in the leaves of selected T1 transgenic lines and wild-type tobacco plants was measured using an ELISA reader (Multiskan EX). Compared with that in the wild-type tobacco plants, the AsA content in the L1, L2, and L4 transgenic plants increased by 1.3-, 1.2-, and 1.32-fold, respectively (Fig. 5F). These results indicate that overexpression of *CvGME* leads to an increase in AsA content in transgenic tobacco plants.

## Discussion

Ascorbic acid (AsA) is a major antioxidant protecting plant cells against reactive oxygen species (ROS) and enhancing plant resistance to biotic and abiotic stresses^29^. GME catalyzes the conversion of GDP-D-mannose to GDP-L-galactose and is a key step in the AsA biosynthesis pathway in higher plants^29^. In the present study, a *GME* homolog was cloned from *C. vestitum* and named *CvGME*. The deduced amino acid sequence of CvGME contains an NAD binding site and a short-chain dehydrogenase catalytic domain, which are well conserved in other species^18,30,31^. The conserved motif of *GME* homologs revealed the evolutionary conservation of *GME* gene function in plants. *CvGME* contains six exons and five introns, similar to most *GME* genes in angiosperms^13^. However, *GME* structure varied greatly in lycophytes, bryophytes, and chlorophytes^13^. Four and six exons were present in *GME* homolog of lycophytes and bryophytes, and one, two, four, six, seven, and eight exons were observed in chlorophytes. *OlGME* isolated from *Ostreococcus lucimarinus* does not have intron structure^13^. These results suggest that *GME* has undergone extensive differentiation in lower plants, but it has been stable in higher plants during evolution. Therefore, it might be a potential phylogenetic marker to decipher the evolution of plants.

The organ-specific expression pattern analysis demonstrated that *CvGME* was highly expressed in plant root and stem; this finding is consistent with the results reported in cotton and *Arabidopsis*^32,33^, suggesting an important role of *CvGME* in the development of stems and roots in *C. vestitum*. The trinscripts of *CvGME* were also detected in leaf, apical bud and flower. These results revealed that *CvGME* was involved in plant growth and reproductive development in *C. vestitum*.

Heterologous expression of *MsGME* from *Medicago sativa* in transgenic *Arabidopsis* increased its AsA content^23^. Similar results were observed in the fruit of transgenic *GME* blueberries^34^. Ascorbate biosynthesis is decreased in *Arabidopsis GME* mutants^22^. Overexpression of *CvGME* led to an increase in AsA content in transgenic tobacco compared with that in the wild-type plants. These results indicate that *CvGME* promotes AsA biosynthesis in tobacco. They also suggest that GDP-d-mannose-3,5-epimerase is a key enzyme in the AsA synthesis pathway, and overexpression of *GME* homolog gene could increase the AsA content in various species.

Exposure to stress leads to the production of large amounts of ROS, and this significantly affects the normal growth and development of plants^29^. To adapt to abiotic stress, plants have evolved a system composed of enzymatic and non-enzymatic antioxidants to neutralize ROS and protect cells from oxidative damage, in which AsA plays a key role as a major non-enzymatic antioxidant molecule^29^. Accumulation of AsA in plants was regarded as an effective way to enhance plant resistance to abiotic stresses^16^. *GME* is a key gene in the AsA biosynthetic pathway in plants. Overexpression of *SlGME1* and *SlGME*2 in tomatoes can improve their tolerance to low-temperature, high-salt, and oxidative stresses^22^. Heterologous expression of *MsGME* from alfalfa in transgenic *Arabidopsis* improves tolerance to drought and salt stress^23^. In this study, the MDA content in the leaves of *CvGME* transgenic tobacco was significantly lower than that of the wild-type under drought stress, suggesting that lipid peroxidation was reduced in *CvGME* overexpressing tobacco compared with that in wild-type. The POD and SOD activities in the leaves of *CvGME* transgenic tobacco were significantly increased under drought stress, compared with those in the wild-type tobacco (Fig. 5). We believe that the increased POD and SOD activities and upregulated *CvGME* expression under drought stress might result from stress-induced stability of *CvGME*.

The present study results indicate that the enhanced drought stress tolerance involved the constitutive expression of *CvGME* in tobacco. Therefore, *CvGME* might be a promising gene for improving drought tolerance in chrysanthemums. However, further studies are needed to explore the expression of genes encoding ROS-related enzymes and drought stress-responsive genes to elucidate the function of *CvGME* in the tolerance of plants to drought and other abiotic stresses.

## Materials and methods

### Ethics statement

All the methods were performed in accordance with relevant guidelines and regulations.

### Plant materials

*Chrysanthemum vestitum* plants were collected from Funiu Mountain in Henan Province, China, and planted in the nursery garden at Northeastern University, China. The Voucher specimens were identified by Mr Zhenhai Wu, a botanist of Northwest A&F University (Yangling, China), and deposited into the Herbarium of Northwest A&F University with the voucher number MYP–20120815 (WUK). Plant tissue samples were collected, immediately frozen in liquid nitrogen, and stored at – 80 °C. Total RNA was extracted using a Plant RNA kit (Omega Bio-Tek, USA) and treated with DNase I (Omega Bio-Tek, USA) to remove genomic DNA. Genomic DNA was isolated from fresh leaves using the cetyltrimethylammonium bromide (CTAB) method described by Couch and Fritz with minor modifications^35^.

### Isolation of the *CvGME* gene

Single-strand cDNA was synthesized from RNA using reverse transcriptase (Thermo Fisher Scientific Inc., Waltham, MA, USA) according to the manufacturer’s instructions. The cDNA and genomic DNA were used as templates for *CvGME* amplification using Pfu DNA polymerase (Takara Tokyo, Japan) with the forward primers 5′-ATGGGAACAACCGGTGAAAC-3′ and reverse primer 5′-TTACTCTTTTCCATCGGCTG-3′ designed according to our transcriptome data of *C. vestitum* (unpublished). The amplified product was purified using a Trace agarose gel DNA recovery kit (Zhongmeitaihe, Beijing, China), linked with the Amp-TOPO vector, and transformed into *Escherichia coli* competent cells according to the method previously described by Hu et al.^36^. Transformed colonies were verified using PCR with restriction digestion and gene-specific primers. Six positive clones were sent to the Zhongmeitaihe Gene Company (Beijing, China) for sequencing.

### Sequence analysis

Putative protein sequences of the GME homologs were retrieved from NCBI database for phylogenetic analysis. Multiple amino acid sequences alignment was performed using DNAMAN 6.0 software. A neighbor-joining tree was constructed using MEGA 6 software with 1000 bootstrap replicates using the Kimura two-parameter distances and pairwise gap deletions.

### Quantitative real-time PCR analysis

Total RNA was extracted from the root, stem, leaf, shoot apical meristem, and fully opened flower to determine the temporal and spatial expression patterns of *CvGME*. Quantitative real-time PCR was performed using the method described by Hu et al.^36^ with special primers of qCvGME-F: 5′-TCATTGATGAATGTGTTGAA-3′, and qCvGME-R: 5′-AGTGATGAGATGGTAAGCAT-3′. *CvACTIN* was used as an internal reference with forward primers: 5′-ATCTGGCATCACACGTTTTACAA-3′, and reverse primer: 5′-TCTCAC-GATTGGCTTTTGGAT-3′. The 2^−ΔΔCt^ method was used to calculate relative gene expression.

### Subcellular localization and transient expression of *CvGME*

The *CvGME* coding sequence without the termination codon was amplified with primers harboring the *Xba* I and *Kpn* I restriction sites (Supplementary Fig. S1) and then inserted into the pBI121-GFP vector to generate the expression vector pBI121-*CvGME*-GFP. The forward primer sequence was 5′-AACTCTAGAATGGGAACAACCGGTGAAAC-3′ and the reverse primer sequence was 5′-ACTGGTACCATACTCTTTTCCATCGGCTG-3′. The subcellular localization of *CvGME* was examined using transient expression in onion epidermis following the method described by Hu et al.^36^. The luminescence of the temporary culture plates was observed under a confocal laser microscope (DMI8; Leica, Germany) at an excitation wavelength of 480 nm.

### Transformation of *CvGME* into tobacco

The pBI121-*CvGME* vector (Supplementary Fig. S2) was transformed into *Nicotiana tabacum* leaf disks using *Agrobacterium tumefaciens* strain EHA105 following the method described by Sparkes^37^ with some modifications to examine its biological function. The leaf disks were cultured in a series of Murashige and Skoog medium (MS) containing kanamycin and rifampicin. Rooted transformants were planted in the soil and grown under long-day (LD) condition (16-h light/8-h dark, at 25 °C). The transgenic plants were identified using genomic PCR with forward primer 5′-ATGGGAACAACCGGTGAAAC-3′ and reverse primer 5′-TTACTCTTTTCCATCGGCTG-3′ using Taq polymerase (TaKaRa Tokyo, Japan). Real-time PCR was used to analyse the expression of *CvGME* under drought stress in wild-type and transgenic tobacco. The two primers used to evaluate *CvGME* expression levels were the same as those used for qRT-PCR. *NtACTIN* was used as the internal control in qRT-PCR and amplified with primers *Ntactin*-F: 5′-CATTGTGCTCAGTGGTGG-3′ and *Ntactin*-R: 5′-AAGGGATGCGAGGATGGA-3′.

### AsA content assay

AsA content in the transgenic and wild-type plants was determined as previously described by Stevens^38^. Briefly, approximately 0.2 g of plant leaves were frozen in liquid nitrogen, ground into powder, and then homogenized in 1 mL of precooled 6% trichloroacetic acid solution. Subsequently, the homogenate was centrifuged at 5331×*g* for 15 min at 4 °C, and 20 μL of the supernatant was mixed with 50 μL dithiothreitol (50 mM) in a 96-well ELISA plate and incubated at 37 °C for 20 min. Thereafter, 10 μL N-ethylmaleimide (0.5%, w/v) was added and incubated at room temperature for 2 min. Next, 80 μL of chromogenic agent was added to the mixture and incubated at 37 °C for 90 min. Finally, the absorbance of the sample was measured at 550 nm using a microplate reader (BioTek, USA).

### Drought stress treatment

Transgenic and wild-type tobacco seedlings (4–5 weeks old) were transferred to plastic culture pots (15 cm in diameter) containing a 1:1 ratio of perlite and turf soil and grown under LD condition, 25 °C day/18 °C night temperature, relative humidity of 70%, and a light intensity of 100 µmol/m^2^ s. Rooted plants at the 6–8 leaf stage were selected for the drought stress treatment. After watering the plants until run-off, the water in the tray was sucked dry to start the test at 25 °C day/18 °C night temperature and 70% humidity. Plants were not watered again for the duration of the drought stress treatment. Three replicates were set up for each experiment. The third and fourth leaves counting from the shoot apex were harvested from wild-type and treated plant, frozen in liquid nitrogen, and stored at − 80 °C after drought stress treatment for 0, 5, and 10 days. Subsequently, AsA, and malondialdehyde (MDA) content, and SOD and POD activity were measured.

### MDA content determination

MDA is one of the most important products of membrane lipid peroxidation in plant membrane systems and indirectly reflects the degree of damage to the plant membrane system and stress resistance^23^. To determine MDA content, 0.1 g leaf tissue was homogenized in 5 mL of 5% (w/v) trichloroacetic acid and centrifuged at 664×*g* for 10 min. Then, 2 mL of supernatant was mixed with 2 mL of 0.067% (w/v) thiobarbituric acid, and this solution was boiled for 15 min and immediately placed on ice. After the mixture was centrifuged for 10 min at 664×*g*, the supernatant was used for MDA measurement following the method described by Ma^23^.

### Determination of SOD and POD activities

SOD and POD are antioxidant enzymes that can effectively reduce ROS production in plants, and they play key roles in plants’ responses to abiotic stress. The activities of SOD and POD in tobacco leaves were measured using an an ELISA reader (BioTek Instruments, Winooski, VT, USA) following the manufacturer’s instructions. Briefly, 0.3 g leaves were ground with 5 mL 0.1 mol/L Tris–HCl buffer (pH 5.8), and the homogenate was centrifuged at 1180×*g* for 5 min to extract the crude enzyme solution. The absorbance of the supernatant was measured at 550 nm for SOD analysis, whereas POD activity was measured according to the method described by Pan et al.^39^.

### Statistical analyses

All experiments were performed in triplicate, and the results are represented as the mean ± standard error of three independent experiments. All data were subjected to analysis of variance using GraphPad Prism 6 software (San Diego, CA, USA). Multiple range tests were used to detect significant differences between means, and statistical significance was defined as *p* < 0.05 or *p* < 0.01.

## Conclusion

The *GME* homolog *CvGME* was obtained from *Chrysanthemum vestitum* plants. The homolog contains an open reading frame of 1131 bp and encodes a predicted protein with 376 amino acids. Overexpression of *CvGME* in tobacco increases the AsA content and effectively enhances drought tolerance. Our results suggest that *CvGME* can effectively enhance the drought tolerance of transgenic tobacco via increased ascorbate accumulation. These results further support the idea that molecular breeding of plants containing *CvGME* may help enhance drought tolerance in other plants, such as chrysanthemums, which is of economic and commercial values.

## Supplementary Information

Supplementary Figures.

## Data Availability

The sequence data obtained in this study are openly available in GenBank of NCBI at https://www.ncbi.nlm.nih.gov/ under the Accession Numbers OL962692 and OM304347.
