# Supplementary material for: Molecular cloning and functional analysis of a Chrysanthemum vestitum GME homolog that enhances drought tolerance in transgenic tobacco
Source: Sci Rep. 2022 Aug 8;12:13551. doi: 10.1038/s41598-022-17815-7 (PMC9360411; doi:10.1038/s41598-022-17815-7)
Supplement: Supplementary file 1 — Supplementary Figures. [file 41598_2022_17815_MOESM1_ESM.docx]

**Molecular Cloning and Functional Analysis of a *Chrysanthemum vestitum* *GME* Homolog that Enhances Drought Tolerance in Transgenic Tobacco.**

Jingjing Li^1^, Hongyuan Xu^1^, Xiaoyu Li^1^, Lijun Wang^1^, Xuan Wang^1^, Yanqing Liu^1^ and Yueping Ma^1^*

^1^College of Life and Health Sciences, Northeastern University, Shenyang, 110004, China

*Correspondence: Yueping Ma, College of Life and Health Sciences, Northeastern University, Shenyang, 110004, E-mail: [mypluna@sina.com](mailto:mypluna@sina.com)

Supplementary legends

Figure S1. The sequence used for generate the expression vector and the restriction sites were marked.

Figure S2.The map of 35S -*CvGME* vector.

**A**


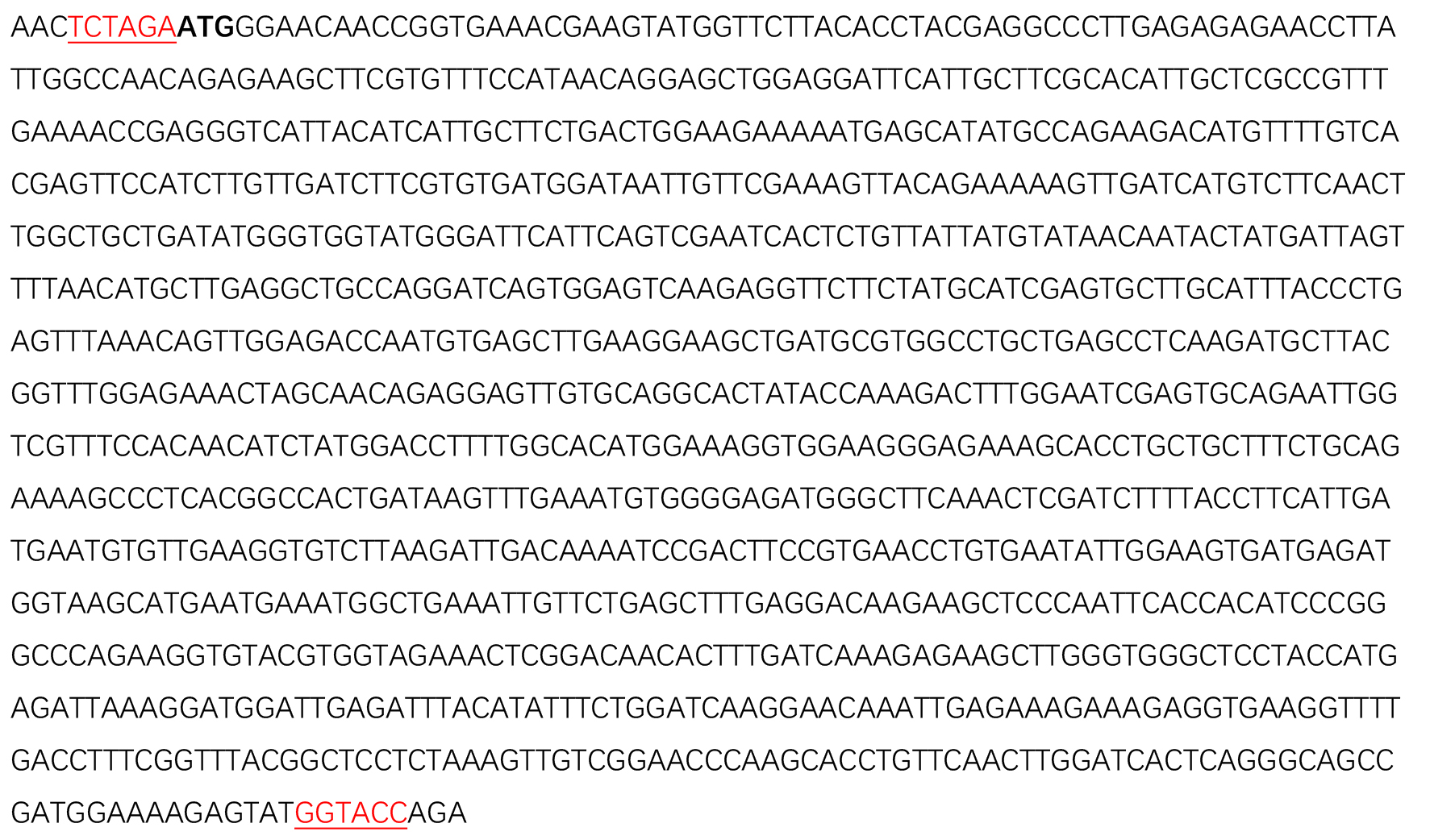


Figure S1. The sequence used for generate the expression vector and the restriction sites were marked.


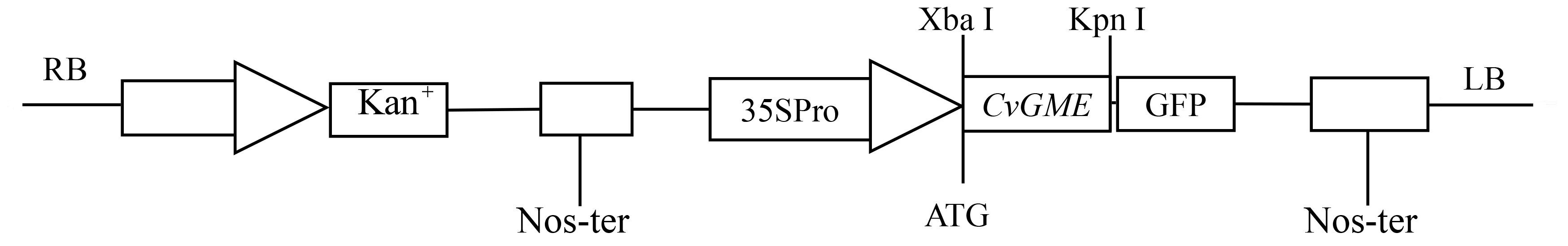


Figure S2.The map of 35S -*CvGME* vector.
